# Pharmacokinetic and pharmacodynamic characterization of a new formulation containing synergistic proportions of interferons alpha-2b and gamma (HeberPAG®) in patients with mycosis fungoides: an open-label trial

**DOI:** 10.1186/2050-6511-13-20

**Published:** 2012-12-28

**Authors:** Yanelda García-Vega, Idrian García-García, Sonia E Collazo-Caballero, Egla E Santely-Pravia, Alieski Cruz-Ramírez, Ángela D Tuero-Iglesias, Cristian Alfonso-Alvarado, Mileidys Cabrera-Placeres, Nailet Castro-Basart, Yaquelín Duncan-Roberts, Tania I Carballo-Treto, Josanne Soto-Matos, Yoandy Izquierdo-Toledo, Dania Vázquez-Blomquist, Elizeth García-Iglesias, Iraldo Bello-Rivero

**Affiliations:** 1 Clinical Investigation Department, Center for Genetic Engineering and Biotechnology, P.O. Box 6332, Havana, Cuba; 2 “Hermanos Ameijeiras” Hospital, Dermatology Service, Havana, Cuba; 3 “Hermanos Ameijeiras” Hospital, Clinical Laboratory Service, Havana, Cuba; 4 Genomics Department, Center for Genetic Engineering and Biotechnology, Havana, Cuba

## Abstract

**Background:**

The synergistic combination of interferon (IFN) alpha-2b and IFN gamma results in more potent *in vitro* biological effects mediated by both IFNs. The aim of this investigation was to evaluate by first time the pharmacokinetics and pharmacodynamics of this combination in patients with mycosis fungoides.

**Methods:**

An exploratory, prospective, open-label clinical trial was conducted. Twelve patients, both genders, 18 to 75 years-old, with mycosis fungoides at stages IB to III, were eligible for the study. All of them received intramuscularly a single high dose (23 × 10^6^ IU) of a novel synergistic IFN mixture (HeberPAG®) for pharmacokinetic and pharmacodynamic studies. Serum IFN alpha-2b and IFN gamma concentrations were measured during 96 hours by commercial enzyme immunoassays (EIA) specific for each IFN. Other blood IFN-inducible markers and laboratory variables were used as pharmacodynamics and safety criteria.

**Results:**

The pharmacokinetic evaluation by EIA yielded a similar pattern for both IFNs that are also in agreement with the well-known described profiles for these molecules when these are administered separately. The average values for main parameters were: Cmax: 263 and 9.3 pg/mL; Tmax: 9.5 and 6.9 h; AUC: 4483 and 87.5 pg.h/mL, half-life (t_1/2_): 4.9 and 13.4 h; mean residence time (MRT): 13.9 and 13.5 h, for serum IFN alpha-2b and IFN gamma, respectively. The pharmacodynamic variables were strongly stimulated by simultaneous administration of both IFNs: serum neopterin and beta-2 microglobulin levels (β_2_M), and stimulation of 2’-5’ oligoadenylate synthetase (OAS1) mRNA expression. The most encouraging data was the high increment of serum neopterin, 8.0 ng/mL at 48 h, not been described before for any unmodified or pegylated IFN. Additionally, β_2_M concentration doubled the pre-dose value at 24–48 hours. For both variables the values remained clearly upper baseline levels at 96 hours.

**Conclusions:**

HeberPAG®possesses improved pharmacodynamic properties that may be very useful in the oncologic setting. Efficacy trials can be carried out to confirm these findings.

**Trial registration:**

Registro Público Cubano de Ensayos Clínicos RPCEC00000130

## Background

Similar to other low molecular weight protein drugs, alpha or gamma interferons (IFNs) have a relatively short serum half-life. Consequently, if vascular retention is considered to be desired for enhanced efficacy, strategies that can improve a drug’s pharmacokinetic (PK) and pharmacodynamic (PD) properties might improve its therapeutic benefits. Novel injectable drug delivery systems have been developed in attempts to improve PK/PD properties of therapeutic peptides and proteins. This can be achieved either by modification of the drug molecule itself (e.g. pegylation) or through a change in formulation (e.g. controlled-release formulations, liposomal preparations) [1].

Another alternative is to potentiate the pharmacodynamics of the therapeutic drug by the combination of two active principles that can act synergistically. This approach could have the same potential advantages of novel delivery mechanisms that include an increased or prolonged pharmacological activity without additional toxicity, less frequent injections, and a better patient’s compliance and quality of life.

IFNs have been widely used in the treatment of human solid and hematological malignancies. However, despite antitumor activity of IFNs is well-known at present, no major advances have been achieved in the last decade. A hopeful option could be the combination of IFN alpha-2b and IFN gamma, two molecules with recognized synergistic antiproliferative effects on several tumor cell lines [2].

Takaoka *et al*. demonstrated in mouse embryonic fibroblasts that IFN gamma response is substantially augmented through autocrine IFN alpha/beta. Additionally, they observed that cross-recruitment and phosphorylation of one of the IFN alpha/beta receptor subunits (IFNAR1) occurred in response to IFN gamma [3]. Similarly, other authors showed that IFNGR1-IFNAR2 (ligand binding domains of both receptor systems) are associated in the presence of IFN alpha and IFN gamma [4]. The physical interaction between both IFN receptor complexes may be the first step for the triggering of intracellular signals that promote the synergism between both IFNs. In the clinics, the peri- and intralesional administration of a new pharmaceutical stabilized formulation containing IFNs alpha-2b and gamma (HeberPAG®), was safe and effective for the treatment of elder patients with advanced, recurrent or resistant to previous treatments basal and squamous cell skin carcinomas [5].

The main objective of this study was to characterize the PK/PD of HeberPAG® in patients with mycosis fungoides. Classical IFN-inducible biological markers, neopterin, β2-microglobulin (β_2_M), and 2’-5’ oligoadenylate synthetase (2’-5’ OAS1), were used as indicators of their pharmacodynamic action. A secondary objective was the registration of adverse events.

## Methods

An exploratory, prospective, open-label clinical trial was carried out at the “Hermanos Ameijeiras” Hospital, Dermatology Service, Havana, Cuba. The protocol was approved by the Ethics Committee of this hospital and by the Cuban Regulatory Authority, the State Center for the Control of Drugs, Equipment & Medical Devices (CECMED, reference number: 999/16.016.09.B). The trial was in compliance with the Helsinki Declaration and its amendments. All patients prior to study enrollment provided their written informed consent.

### Subjects

Twelve patients, both genders, 18 to 75 years-old, with clinically and histologically proven mycosis fungoides, at stages IB to III, were recruited for the study. Other eligibility criteria included a measurable disease, a life expectancy of at least 24 weeks, Karnofsky’s index ≥ 60%, with more than 1 month of previous disease specific treatments or more than 3 months in case of steroid use. Patients also had adequate hematological, hepatic, and renal function. Exclusion criteria were other uncompensated chronic diseases or neoplasias, pregnancy or nursing and severe psychiatric dysfunction.

### HeberPAG formulation

A stabilized formulation containing 3.5 x10^6^ IU of a synergistic combination of human recombinant, produced in *E. coli*, IFNs alpha-2b and gamma (HeberPAG®, Heber Biotec, Havana, Cuba), was used for all cases. Each vial also contains 18.87 mg sodium phosphates, 13 mg Dextran-40, 0.24 mg kalium phosphate, 16.59 mg sodium chloride, 0.18 mg kalium chloride, 5.0 mg manitol, 5.0 mg saccharose, and 5.5 mg human albumin. This lyophilized powder formulation was reconstituted with 2 mL bacteriostatic water for injection.

### Study design

The study was designed to calculate pharmacokinetic and pharmacodynamic parameters after a first single high dose of HeberPAG®. Each patient received, intramuscularly, in the gluteus region, a single 23 x10^6^ IU HeberPAG® dose, chosen to obtain detectable serum levels of both IFNs. Antipyretic medication was given orally at the same time as the HeberPAG® injection and every 4 hours thereafter, up to 12 hours or more if needed in order to mitigate the expected IFN-dependent flu-like syndrome. Patients were hospitalized during the first 96 hours after the injection under strict medical supervision. After this period the study continued to evaluate efficacy and safety of this product in the same group of patients. Then, they received 11 x10^6^ IU twice a week during one year.

### Laboratory evaluations

Blood samples for serum IFN alpha-2b and IFN gamma concentration determinations were collected by venipuncture before and 1, 3, 6, 12, 24, 48, 72, and 96 hours after injection. Pharmacodynamics was assessed by serum neopterin and β_2_M concentrations at the same times and by the induction of 2’-5’ OAS1 mRNA expression before and at 6, 12, 24, 48, 72 and 96 hours. Routine hematological and biochemical determinations were taken as safety variables, every 24 hours during the first 96 hours. These included hemoglobin, hematocrit, leukocytes and platelets counts, transaminases, bilirubin, creatinine and urea. Patients were regularly checked for vital signs and symptoms during the whole hospitalization.

Vacutainers were used to collect blood samples to determine serum concentrations and biochemistry (8.5 mL Z Serum Sep, Greiner bio-one) and for hematology analysis (4 mL K3E K3EDTA, Greiner bio-one). Blood samples for total RNA purification were collected in PAXgene Blood RNA Tubes (2.5 mL, QIAGEN, US).

Serum IFNs, neopterin and β_2_M levels were measured using commercially available kits according to the manufacturer’s instructions using sera stored at −80°C until be tested. IFN alpha-2b and IFN gamma were quantified in serum with high sensitivity enzyme immunoassay (EIA) kits specific for IFN alpha (Catalogue: BMS216CE, Bender MedSystem, GMBH) or IFN gamma (Catalogue: BMS228CE, Bender MedSystem, GMBH), respectively. Neopterin was determined by a commercial EIA kit (HENNING test, BRAHMS Diagnostica GmbH, Berlin, Germany) as well as serum β_2_M (Quantikine® IVD®, R&D System, Inc, Minneapolis).

Quantification of OAS1 mRNA levels was performed using the Real-Time Polimerase Chain Reaction (qPCR) method. Total RNAs were obtained by PAXgene purification protocol (PreAnalytiX/Qiagen, US). RNA quality was checked in a Spectrophotometer Nanodrop 1000 (ThermoScientific, US), reporting a 260/280 nm OD relation between 1.7 and 2.2. Agarose electrophoresis allowed to visualize 28S rRNA and 18S rRNA bands in a proportion higher than 1.5. Complementary DNA (cDNA) was used as template in qPCR experiments; the synthesis was carried out using Superscript II RT kit and protocol (Invitrogen, US) from total RNA samples at each time point. qPCR experiments were performed in 20 μL using ABsolute QPCR SYBR Green Mixes (ThermoScientific, ABgene, UK) and 0.3 μM of primers for amplification of OAS1 target gene (F: 5’ AGCCTCATCCGCCTAGTCAA 3’; R: 5’ CTCGCTCCCAAGCATAGACC 3’) and reference genes GAPDH (F: 5’ CCATGGGTGGAATCATATTGGA 3’; R: 5’ TCAACGGATTTGGTCGTATTGG 3’) and HMBS (F: 5’ GGAATGTTACGAGCAGTGATGC 3’; R: 5’ CCTGACTGGAGGAGTCTGGAGT 3’). All of them were designed on the basis of the GenBank database information using primer3 software [6]. All experiments were in triplicates, rendering amplifications curves between cycles 15 and 30 in RT™Cycler equipment (Capitalbio, China) with a standard program of 15 min at 95°C for enzyme activation followed by 40 cycles of 15 s at 95°C, 30s at 60°C and 30s at 72°C. Capitalbio software reports of Ct values, as the 2nd derivative maximum, and values of fluorescence per cycle, which were used for efficiency calculation by LinReg software (version 11.3, 2009), were used for calculations of relative OAS1 gene expression, at each time point respect to time 0 h, using REST-MCS (version 2, 2006) software [7], after the normalization with reference genes.

Hematological counts and blood chemistry were done according to usual clinical laboratory procedures, using advanced automated analyzers.

### Data analysis

The drug disposition data analysis was performed per patient by a non-compartmental method with a combined linear/log - linear trapezoidal rule approach. The linear trapezoidal rule was used up to peak level and the logarithmic trapezoidal rule thereafter. The first-order rate constant associated with the curve terminal (log linear) portion (λ) and terminal half-life (t_1/2_) were estimated by linear regression of the included terminal data points. Time-to-peak values (Tmax) were determined directly from the experimental data as the time of maximum observed level (Cmax) considering the entire curve. Area under the serum concentration-time curve from 0 to 96 hours (AUC_96_) was calculated using the linear/log linear trapezoidal rule. Mean residence time (MRT) was also calculated using the moments of the drug disposition curve. Parameters that were extrapolated to infinity, such as AUC (area under disposition curve) and AUMC (area under first moment of the disposition curve) were computed based on the last predicted value from the linear regression performed to estimate λ and t_1/2_. Some similar kinetic parameters were estimated for the pharmacodynamic markers, corrected for baseline values, neopterin and β_2_M in order to describe the kinetic behavior of the IFN-induced immunological response: Rmax (maximum response), T(Rmax) (time to reach maximum response), λ effect (effect dissipation constant), t_1/2_ effect (effect half-life), AUEC (area under the effect curve), MET (mean effect time) [8]. The WinNonlin professional software (Version 2.1, Pharsight Inc., 1997, NC, USA) was used for all these purposes. A descriptive statistic was done using SPSS for Windows version 15.0.

## Results

Twelve patients, 7 females and 5 males, with a disease stage IIA and III, were recruited. They were between 33 and 74 years-old (mean: 53.3 yrs), with a mean corporal surface of 1.79 m^2^ (range: 1.46 – 2.28). Three clinical variants of mycosis fungoides were represented; 6 patients had plaques, 2 had erythroderma, and the rest had atypia of the epidermis. Only five patients had received prior systemic antitumor therapy, four of them IFN alpha and three methotrexate. Other previous treatments (cyclosporine, steroid cream, radiotherapy) were received by a single patient each one. Initial mean LDH was 216 U/L (range: 69 – 355). Most of the patients complied with the evaluations as previewed, except for patient No.8 whose serum samples were not available at 72 and 96 hours.

### Pharmacokinetic analysis

**Figure 1:**
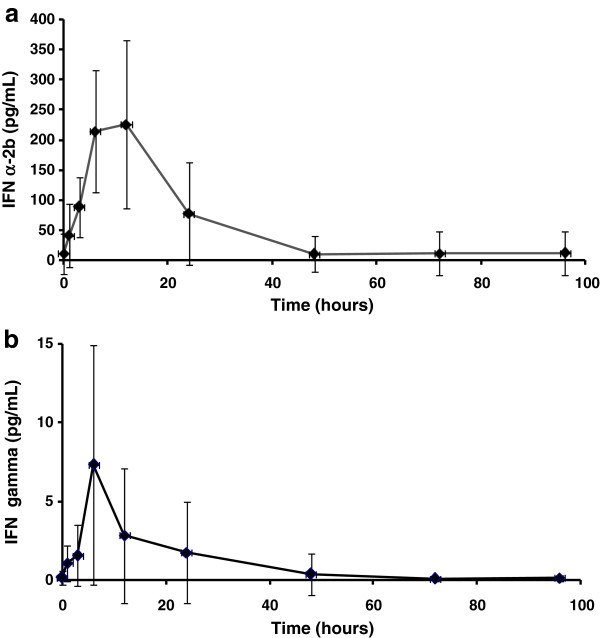
**Interferon concentrations in serum.** Legend: Data correspond to 12 patients with mycosis fungoides who received 23 × 10^6^ IU of HeberPAG®. Each point represents the average and standard deviation of (**a**) IFN alpha-2b and (**b**) IFN gamma levels, both measured by EIA

Except for two patients, who had 7.1 and 114 pg/mL of IFN alpha-2b, all had undetectable or very low endogenous pre-dose serum IFN concentrations (median = 0 pg/ml). The average concentration profiles obtained for both IFNs are showed in Figure 1. IFN alpha-2b concentrations started to increase notably from the first hour post-injection. At 3 hours, more than half of the patients reached 100 pg/mL. Maximum values (> 200 pg/mL) were obtained at 6–12 hours in most of the cases (Figure 1a). Patient No. 12, who had the higher baseline value, reached 440 pg/mL at 12 hours. Increments in IFN gamma levels were more discreet with a peak at 12 hours post-injection, where 5 patients reached 15 pg/mL or more (Figure 1b). A pronounced drop of IFN concentrations was detected since 24 hours. At 48–96 hours after the injection, these values had returned near to the initial values, so the AUC_96_ obtained covered more than 95% of the AUC extrapolated to infinite.

Table 1 shows the results of the pharmacokinetic parameters calculated from the above commented profiles. A high patient -dependant variability is observed, mainly with IFN gamma (see SD). Additionally, an elevated distribution volume and fast blood clearance of both IFNs was obtained (data not shown).

**Table 1:** Pharmacokinetic parameters calculated from the IFN alpha-2b and IFN gamma concentrations in serum (N = 12)

**Parameter**	**IFN alpha-2b**	**IFN gamma**
Cmax (pg/mL)	263 ± 129	9.3 ± 7.0
Tmax (h)	12.0 ± 6.0	6.0 ± 0.8
λ (h^-1^)	0.16 ± 0.06	0.07 ± 0.05
t_1/2_ (h)	4.9 ± 1.4	13.4 ± 27.1
AUC (pg.h/mL)	4483 ± 4485	87.5 ± 89.9
MRT (h)	13.9 ± 7.9	13.5 ± 8.2

Data are reported as mean ± standard deviation, except for Tmax expressed as median ± quartile range.

### Pharmacodynamic analysis

**Figure 2:**
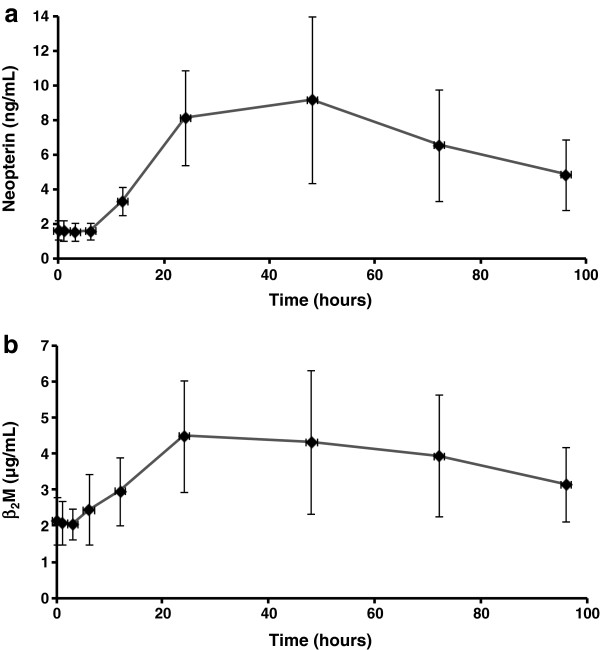
**Pharmacodynamic markers neopterin and β2-microglobulin in serum.** Legend: Data correspond to 12 patients with mycosis fungoides who received 23 **×** 10^6^ IU of HeberPAG® at time 0. (**a**): Average neopterin concentration, measured by EIA. (**b**): Average β_2_M concentration, measured by EIA. Standard deviations are also showed at each time

The time courses for the induction of neopterin and β_2_M are showed in Figure 2. Both variables were strongly stimulated by simultaneous administration of both IFNs with highest inductions around 24–48 hours after intramuscular administration. HeberPAG® induced an average increment of serum neopterin at 48 hours about approximately six times, until 9.6 pg/mL compared to pre-dose values (Figure 2a). Individually, five patients surpassed 10 pg/mL at this time. Figure 2b shows that mean serum β_2_M peaked around the double from baseline at 24–48 hours. For both variables the values remained clearly upper baseline levels until 96 hours. Notably, neopterin remained three times superior to pre-dose value at the end of the sampling period.

A pharmacokinetic-like analytical procedure was carried out with both variables (Table 2). Since patients could have pre-treatment levels of these biological markers, a more real interpretation of kinetics has to be showed as fold increases over baseline. For neopterin Rmax was 8.0 ng/mL, with a t_1/2_ effect = 40 hours and a MET around 80 hours. For β_2_M, T(Rmax) was 2.7 μg/mL, and the effect dissipation phase occurred similarly slow compared to neopterin. A high intra-patient variability was again evidenced.

**Table 2:** Descriptive parameters of the kinetics of serum Neopterin and serum β2-microglobulin increments (N = 12)

**Parameter**	**Neopterin**	**β2-microglobulin**
Rmax	8.0 ± 4.2 ng/mL	2.7 ± 1.4 μg/mL
T(Rmax)	48 ± 24 h	24 ± 24 h
λ effect	0.02 ± 0.01 h^-1^	0.02 ± 0.01 h^-1^
t_1/2_ effect	40.3 ± 12.9 h	37.6 ± 15.9 h
AUEC	661 ± 322 ng.h/mL	195 ± 117 μg.h/mL
MET	80.6 ± 17.4 h	74.8 ± 21.9 h

Data are reported as mean ± standard deviation, except for T(Rmax) expressed as median ± quartile range.

**Figure 3:**
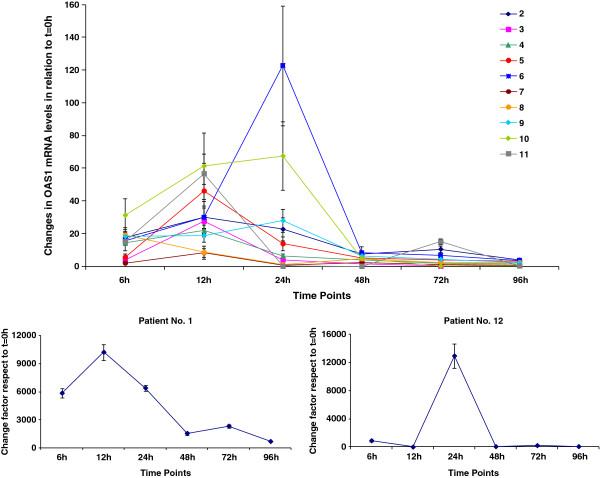
**Individual relative 2’-5’ OAS1 mRNA expression.** Legend: Data correspond to 12 patients with mycosis fungoides who received 23 **×** 10^6^ IU of HeberPAG® at time 0. Each point represents the relative amount and its associated standard error of OAS1 gene expression at each time respect to time 0, after the normalization with reference genes GAPDH and HMBS. Patients No. 1 and 12 are separately graphed since their very high specific mRNA expression

Since the individual induction of the enzyme 2’-5’ OAS1 was measured through the relative amount of its mRNA expression using the Real-Time PCR method, an important variability could be expected. Therefore, parameters could not be rigorously calculated from the experimental data and the analysis was essentially qualitative. Figure 3 shows individual relative OAS1 mRNA levels at each time point with respect to time zero, normalized with GAPDH/HMBS levels. A single intramuscular dose of 23 x10^6^ IU of HeberPAG® resulted in high increases of this variable with regard to their pre-dose values in all patients, in a factor between 8 and 122 times in ten patients, primarily within the first 24 hours after dosing. The expression levels return to initials by 96 hours in most of the patients.

### Safety data

Adverse events were checked during the whole study. All the patients presented at least one event, mostly flu-like symptoms caused by IFN. The most frequent events were fever (100%), malaise (91.7%), headache (58.3%), tachycardia (41.6%), leucopenia (33.3%), chills (33.3%) and anemia (25%). Anorexia, arthralgias, increase of transaminases and myalgias were recorded in two patients. Most of the events (92.3%) were considered mild, none severe, being well controlled.

## Discussion

Treatment with unmodified IFNs for several malignancies and chronic viral affections requires frequent injections (e.g., daily or three times weekly) over the course of therapy due to the molecule’s short circulating half-life in humans. Increase of doses and prolonged therapy could favor a better clinical response but it could also lead to magnify adverse events. Besides, patient’s compliance under long-term dosing regimens is difficult to preserve. The development of more slowly cleared IFNs has allowed to reduce dosing frequency and to enhance response rates in patients with chronic hepatitis C [9]. However, modified high molecular IFNs could have more difficulties to penetrate the tumor niches bearing a reduction in their antitumor effects. A significant reduction of in vitro biological activity has been demonstrated for pegylated IFNs due to non optimal interaction with IFN receptor [10].

Therefore sustained full IFN-receptor interactions with more potent antiproliferative activity are desired in the treatment of cancer. This is possible to obtain combining IFN-alpha and IFN gamma that synergize for their biological activities. HeberPAG® is a new formulation containing a mixture of recombinant IFN alpha-2b and IFN gamma at synergistic proportions. This formulation was created to improve antiproliferative and other biological effects of conventional IFNs with an adequate tolerability leading to administer fewer doses similar to others currently available therapy. This is the first PK/PD study in humans with this variety of IFN formulation.

The pharmacodynamic variables measured in this trial to characterize HeberPAG® formulation are well-known IFN-induced genes, classical surrogate markers of IFN biological actions. Neopterin is a sensitive marker of T helper 1-cell immune response, because it is primarily produced by monocytes/macrophages after activation by IFNs and augments the production of tumor necrosis factor in peripheral blood mononuclear cells [11]. Beta2-microglobulin plays an important role in the tumor growth control and metastases [12]. Progression of the cell cycle is mediated by 2’- 5’OAS levels stimulated by IFN [13]. Additionally, antiviral effects subsequent to IFNs addition are initiated by synthesis of 2’- 5’oligoadenylates that activate an endoribonuclease to cleave double-stranded viral RNA [14].

The most remarkable result was the six-fold increase of serum neopterin concentrations respect to basal value. This high increment induced by HeberPAG® has not been described before in the literature with any subtype or variant of IFN, even for pegylated forms [15-20]. The induction by pegylated IFN-alpha could only approximately tripled the neopterin basal values as maximum 48 hours after injection as reported [17,19,20]. In the case of PEG-IFN beta although half-life was greatly extended by pegylation, the neopterin response was not affected [18]. On the other hand, two times higher levels than baseline were recorded for serum β_2_M 24–48 hours after injection, superior to those increments detected by other authors, which were around 60% with natural or pegylated IFN-alpha [19-21]. For both pharmacodynamic markers their more slow return to initial levels has not been observed with conventional IFN in the reports above-cited. This last result could lead to space the dosage interval for the IFN mixture formulation until twice or once a week. Recent data obtained with the same markers but in healthy male volunteers [article in preparation] emphasize that possibility. Efficacy trials evaluating these frequencies of administration in several oncologic pathologies are under development.

After the single intramuscular injection, 2’- 5’-OAS1 mRNA levels were extensively increased which was also recently found in a group of healthy male volunteers who received 24.5 × 10^6^ IU of a similar formulation. Although mRNA induction does not ensure the presence of active protein, it has been reported that 2’-5’ OAS enzyme activity in the serum of IFN-treated patients appears to increase since the first 6 hours and maintains elevated levels for as long as 4 to 8 months after the initiation of daily IFN treatment [22].

At molecular level this beneficial pharmacodynamic effects could be explained by synergistic effects in the expression and activation of several genes regulated by both IFNs [23].

Concerning pharmacokinetics, no interferences by simultaneous administered IFNs were observed in their typical similar serum profiles. Parameters as Tmax and t_1/2_ were within the reported ranges for these conventional IFNs after systemic administration either in patients or healthy volunteers even considering the expected high variability [24-27]. For IFN alpha-2b Cmax was also very similar to a previous report ours in healthy male volunteers [21].

Flu-like symptoms and other clinical and laboratory adverse events associated with HeberPAG® have been previously reported for recombinant IFN treatment [28]. Fever began 2 to 4 hours after intramuscular administration and peaked at 6 to 12 hours coincident with maximum IFN serum levels. However, the mechanisms of fever induction appear to be different between IFNs. IFN-alpha has been shown to be intrinsically pyrogenic and the body temperature rise is related to the interaction of IFN alpha to hypothalamic μ-opioid receptors [29]. Meanwhile the administration of IFN gamma stimulates the release of other lymphokines such as interleukin-1 [30], an endogenous pyrogen [31].

## Conclusion

The co-administration of IFN alpha-2b and IFN gamma with potent synergistic actions will allow us to obtain a more favorable pharmacodynamics introducing new promissory perspectives in the use of IFNs to treat several malignancies. Efficacy trials can be carried out to ratify the obtained results.

## Competing interests

Authors YGV, IGG, ACR, ADTI, YDR, YIT, DVB, EGI and IBR are employees of the Center for Genetic Engineering and Biotechnology (CIGB), Havana network, where IFN alpha-2b and IFN gamma are produced and the new synergistic formulation (HeberPAG®) was developed. The rest of the authors have no competing interests at all. The study was financed by Heber Biotec, Havana, Cuba (product, reagents), and the Ministry of Public Health of Cuba (hospital facilities and general medical care of the patients).

## Authors’ contributions

YGV designed, coordinated and performed the study, analyzed the results and revised the manuscript. IGG participated in the analyses of results and wrote the manuscript draft. SECC (main clinical investigator), EESP and CAA took care of patient recruitment, management, clinical examinations, and follow-up. ACP carried out EIA determinations. EGI participated in the study design and ADTI achieved the statistical analysis. MCP, NCB, TICT and JSM carried out clinical laboratory determinations. YDR assisted as study monitor. YIT and DVB did the Real time PCR method. IBR conceived the study and took part in the design, results analysis and manuscript writing. All authors read and approved the final manuscript.

## Pre-publication history

The pre-publication history for this paper can be accessed here:

http://www.biomedcentral.com/2050-6511/13/20/prepub
